# Effect of climate change on stormwater runoff characteristics and treatment efficiencies of stormwater retention ponds: a case study from Denmark using TSS and Cu as indicator pollutants

**DOI:** 10.1186/s40064-016-3103-7

**Published:** 2016-11-15

**Authors:** Anitha Kumari Sharma, Luca Vezzaro, Heidi Birch, Karsten Arnbjerg-Nielsen, Peter Steen Mikkelsen

**Affiliations:** 1 Department of Environmental Engineering (DTU Environment), Technical University of Denmark, Bygningstorvet, Building 115, 2800 Kgs. Lyngby, Denmark; 2 Resources ID, Tjørnevej 3C, 3480 Fredensborg, Denmark

**Keywords:** Climate change effects, Stormwater runoff quality, Dynamic model, Treatment, TSS, Copper

## Abstract

This study investigated the potential effect of climate changes on stormwater pollution runoff characteristics and the treatment efficiency of a stormwater retention pond in a 95 ha catchment in Denmark. An integrated dynamic stormwater runoff quality and treatment model was used to simulate two scenarios: one representing the current climate and another representing a future climate scenario with increased intensity of extreme rainfall events and longer dry weather periods. 100-year long high-resolution rainfall time series downscaled from regional climate model projections were used as input. The collected data showed that total suspended solids (TSS) and total copper (Cu) concentrations in stormwater runoff were related to flow, rainfall intensity and antecedent dry period. Extreme peak intensities resulted in high particulate concentrations and high loads but did not affect dissolved Cu concentrations. The future climate simulations showed an increased frequency of higher flows and increased total concentrations discharged from the catchment. The effect on the outlet from the pond was an increase in the total concentrations (TSS and Cu), whereas no major effect was observed on dissolved Cu concentrations. Similar results are expected for other particle bound pollutants including metals and slowly biodegradable organic substances such as PAH. Acute toxicity impacts to downstream surface waters seem to be only slightly affected. A minor increase in yearly loads of sediments and particle-bound pollutants is expected, mainly caused by large events disrupting the settling process. This may be important to consider for the many stormwater retention ponds existing in Denmark and across the world.

## Background

Stormwater runoff from impervious areas such as roads, roofs and parking lots is increasingly catching attention due to impacts like increased frequency of flooding, deterioration of water quality in receiving waters and risk to the downstream ecosystems (e.g. Eriksson et al. 2007; Kayhanian et al. 2008; McQueen et al. 2010; Milly et al. 2002; Walsh et al. 2012). Stormwater runoff contains a range of pollutants in dissolved and particle bound forms. The adverse effect of a substance depends on properties like persistence, toxicity and bioaccumulation. These features are affected by the partitioning of the substance between the dissolved and particulate phases as well as by the exposure and uptake mechanism of the type of flora/fauna considered. For example, hydrophobic organic compounds and heavy metals can sorb to suspended solids carried with stormwater runoff, accumulate in sediments of receiving waters and negatively impact benthic organisms (e.g. Nakajima et al. 2006; Gardham et al. 2014), and compounds freely available in the water phase such as dissolved copper can lead to direct toxic effects to water living organisms (e.g. Allen and Hansen 1996; Ma et al. 2002). Stormwater pollutants are regulated by different legislation around the world, e.g. the European Water Framework directive and the Environmental Quality Standards directive (European Commission 2000, 2008) or the U.S. Clean Water Act (US EPA 2016). Management of stormwater quality is thus an essential part of strategies to improve the environmental status of natural waters. Most design practices for constructing stormwater management facilities are, however, based on historical climate conditions. Climate predictions show that the future conditions will be different from those of the past, with an expected increase in the frequency of heavy rainfall events and droughts in the future in some regions (Parry et al. 2007; IPCC 2012). A reassessment of the feasibility of the adopted stormwater management strategies is therefore appropriate. Water quantity effects of climatic change related to stormwater runoff and urban drainage systems have been studied (e.g. Semadeni-Davies et al. 2008a; Arnbjerg-Nielsen et al. 2013), whereas effects of climatic change on urban emissions including water carried pollutants from stormwater systems have so far received little interest.

Stormwater retention ponds, also called wet detention ponds, are among the most widely used Best Management Practices (BMP) for stormwater management (USEPA 1999; Scholes et al. 2003). Sedimentation is the primary removal mechanism in wet detention ponds for several stormwater pollutants, but depending on the substance properties, processes like adsorption, microbial degradation, and volatilization can also be important (Scholes et al. 2008). Removal of suspended solids (TSS) and other pollutants associated with solids via sedimentation depends mainly on the hydraulic retention time (HRT), with longer HRT leading to higher removal rates (e.g. USEPA 1999; Vollertsen et al. 2007). One of the major characteristics of stormwater runoff is the high temporal and spatial variability of hydraulic flow and pollutant concentrations (e.g. He et al. 2010; Gnecco et al. 2005; Jacobsen 2011; Wium-Andersen et al. 2011). Various relationships have been proposed in the literature to link stormwater quality to different catchment attributes such as land use, rainwater quality and traffic loads (especially for roads and highways), and to forcing functions such as rainfall intensity (when the release of pollutants from a surface is assumed to be linked to the raindrop energy), runoff volume (when pollutant release is assumed to be dependent on the runoff stress on the catchment surface), antecedent rainfall volume and antecedent dry periods (ADP) (e.g. Vaze and Chiew 2003; Ouyang 2003; Gnecco et al. 2005; Goonetilleke et al. 2005; Obropta and Kardos 2007; He et al. 2010, 2011). The latter is commonly used to estimate the mass of particulate pollutants that are available on the catchment surfaces, which, as confirmed by the measurements carried out by Vaze and Chiew (2002), increases during dry weather with an asymptotic behavior. The importance of ADP is magnified in areas characterized by long dry periods and high intensity rainfall events (Sabin et al. 2005).

Increased intensity of heavy rain storms and longer dry weather periods are some of the important expected climate change effects. For a return period of 20 years this is a global trend (IPPC IPCC 2012) and Sunyer et al. (2014a, b) show that for Denmark increasing occurrences of precipitation extremes for short time scales are likely to occur for return periods of 0.2 years and higher. These changes may affect stormwater runoff quality (e.g., Wilson and Weng 2011; He et al. 2011) and the efficiency of stormwater treatment systems, because longer dry weather periods may lead to increased build-up of sediments on catchment surfaces and thus higher concentrations and load pulses in the runoff. This may be exacerbated by the more intense heavy rain storms. High flows may furthermore disrupt the settling process and shorten the HRT of stormwater retention ponds during extreme conditions, which may lead to higher pollution concentrations and loads being emitted to the environment. These effects should therefore be taken into account during the selection of stormwater pollution control strategies as part of surface water protection plans, so that climate-change resilient solutions can be implemented (e.g. Charlesworth 2010). This can be achieved by applying integrated stormwater quality models in combination with analysis of climate change scenarios. Semadeni-Davies et al. (2008b) and Hathaway et al. (2014) conducted such studies focusing on the hydrological behaviour of stormwater control measures.

The aim of this study was to investigate the potential effect of climate changes on the quality of stormwater runoff and on the treatment efficiency of a stormwater retention pond in Denmark by applying an integrated stormwater quality model for long term simulations with a realistic climate change scenario as input. The analysis focused on total suspended solids (TSS), and copper (total and dissolved Cu) loads and concentrations in runoff from a catchment as well as in the outlet of a stormwater retention pond. These were selected among a wider range of stormwater priority pollutants as indicators of physical pollutants and micropollutants (MP) that can potentially be removed via settling. TSS is closely associated with immediate as well as accumulated aesthetic and environmental effects. Cu is ubiquitous in stormwater runoff and available in both dissolved and particulate phases, where especially the dissolved phase causes acute toxicity to aquatic life (Eriksson et al. 2007; Ingvertsen et al. 2011) and therefore is regulated (European Commission 2000, 2008). The stormwater pollutant fluxes were estimated by using the integrated dynamic stormwater quality model presented by Vezzaro et al. (2012a). The potential effect of climate changes on the rainfall pattern and consequently on the stormwater runoff quality and treatment efficiency of stormwater retention ponds was evaluated by applying two 100-year synthetic rain series, where one represented the current climate conditions and the other represented the climatic conditions expected 100 years into the future.

## Methods

### Overall approach to the investigation

**Fig. 1:**
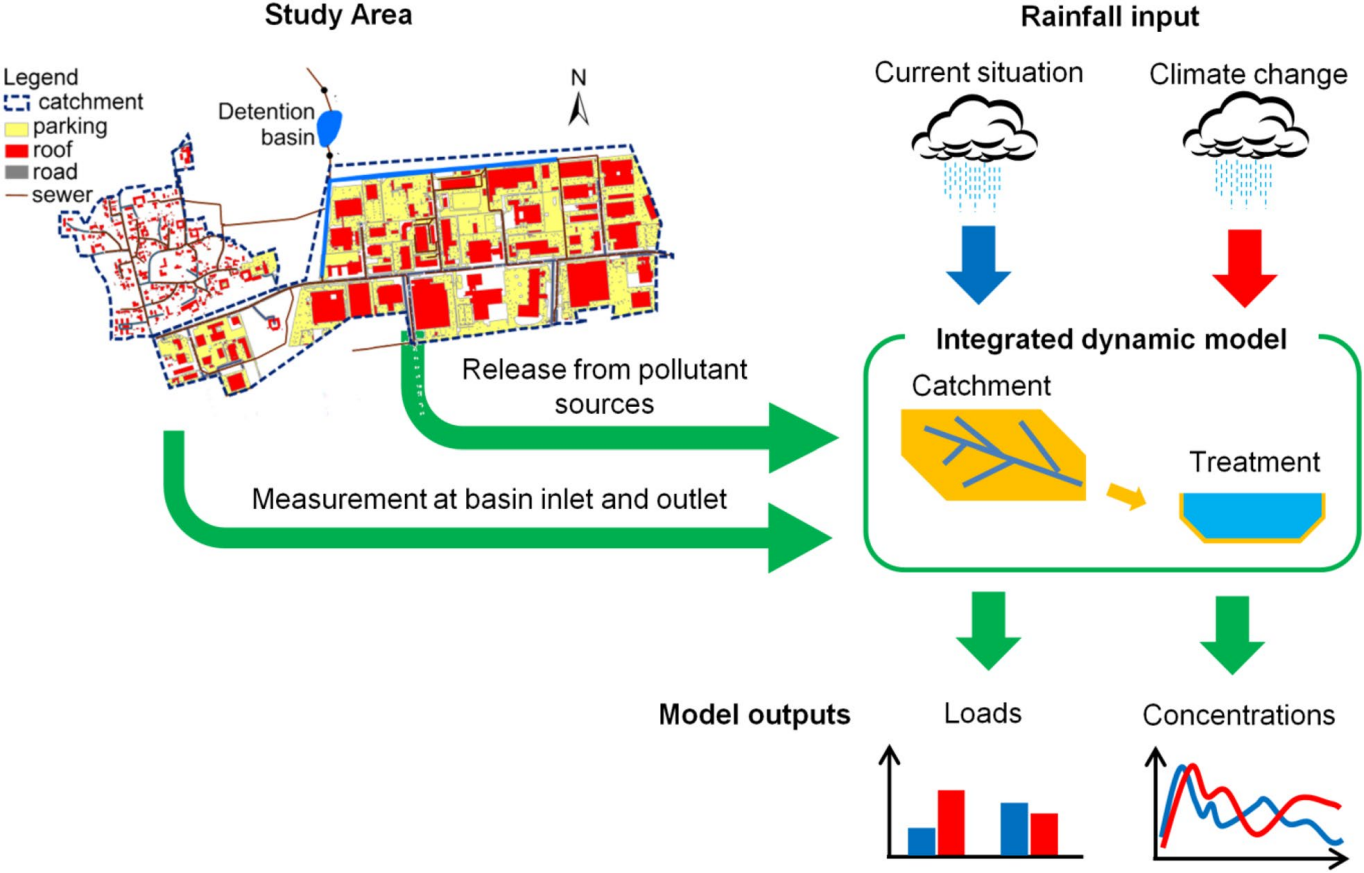
Schematic representation of the data and tools used in the study

**Fig. 2:**
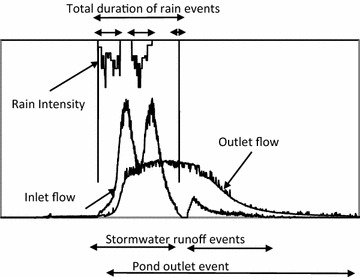
Definitions of a rain event, a stormwater runoff event and a pond discharge event

To investigate the effect of climate changes on the quality of stormwater runoff, the existing stormwater system was subdivided into two subsystems: stormwater catchment and stormwater retention pond (Fig. 1). These two subsystems were modelled by using an integrated model, which employed rainfall data as a major forcing function to the system. The analysis focused on the major outputs from the model, such as TSS and Cu loads and concentrations. Given the long time interval covered by the analysis (up to 100 years, see section on rainfall time series below), the model outputs were analysed to provide statistical information, which was expressed both on a yearly and on an event basis. As the modelled processes (rainfall, runoff generation, transport and stormwater treatment in the pond) have different temporal scales, different definitions of “events” were adopted (Fig. 2): *rain events* [separated by an ADP > 1 h, corresponding to the definition used within urban hydrology in Denmark (Jørgensen et al. 1998)], *stormwater runoff events* (separated when runoff from the catchment equalled the catchment baseflow for more than 1 h), and *pond discharge events* (separated when the pond discharge equalled the baseflow for more than 1 h). These definitions imply that some events can be lumped according to the observed point of the system (i.e. several rain events can generate a single runoff event, and different runoff events can be lumped into a single pond discharge event). The event definitions were used to calculate Event Mean Concentrations (EMC) at the pond inlet and outlet. The EMC of an event is calculated as the ratio between the total mass discharged during an event and the total volume of the event.

### Catchment description and data characterizing the current situation

The study was conducted at the Hersted Industripark catchment in Albertslund, Denmark, which covers 95 ha of industrial and residential areas. Surface runoff from the catchment is collected by a separate stormwater system and led to a stormwater retention pond (Basin K) with a 6400 m^2^ surface area and an average dry weather depth of 0.8–1 m. The outlet from the pond discharges to the Harrestrup stream, for which the surrounding municipalities have a vision to improve the water quality and establish recreational riparian areas (Visionsplan 2007). This will require a substantial reduction of the pollution discharge from this and several other catchments, and the catchment thus exemplifies areas where stormwater pollution control strategies are under development and where the potential impacts of climate change have so far not been considered.

The collected field-data used for calibrating the model included: rainfall, stormwater runoff quantity (flow) and quality (concentrations) from the catchment (inlet to the pond), and quantity and quality of the pond discharge. Inlet and outlet flow data were collected from September 2009 to July 2011 using Nivus PCM 4 flow meters with ultrasonic sensors. Rainfall data were retrieved from a rain gauge located in Albertslund (3 km south-west from the catchment), belonging to a network of the Danish Water Pollution Committee, operated by the Danish Meteorological Institute (Jørgensen et al. 1998). 10 stormwater runoff events (generated by 20 rain events) were sampled to monitor the stormwater quality in the pond inlet and outlet during May 2010–May 2011. At the inlet, samples were taken by volume proportional sampling using an ISCO 3700c autosampler coupled to the flow meter. Sub-samples of 50 mL were taken using an interval of 30–50 m^3^ runoff (before each event, the interval was decided depending on the weather forecast), and 6 sub-samples were composited for each sample. At the outlet, samples were taken by time proportional sampling using a Bühler 1029 autosampler from Hach Lange and sub-samples of 50 mL were taken with 20–40 min intervals compositing 6 sub-samples for each sample. The collected samples were analysed for TSS and total and dissolved Cu (Cu_tot_ and Cu_diss_). TSS was analysed by filtering the sample through 1.5 μm Whatman™ 934-AH™ glass microfiber filters and drying the filtrate at 105 °C. Total and dissolved (0.45 μm filter) Cu were analysed using inductively coupled plasma optical emission spectroscopy (ICP-OES).

### Integrated dynamic simulation model

Fluxes of stormwater pollutants were estimated by using the dynamic integrated, lumped conceptual stormwater quality model presented by Vezzaro et al. (2012a). This model combines a catchment submodel (Vezzaro and Mikkelsen 2012) with a stormwater treatment unit submodel for micropollutants (STUMP—Vezzaro et al. 2010). The catchment submodel estimates stormwater runoff flow based on a non-linear reservoir approach. Stormwater runoff quality is estimated based on an accumulation-washoff process, where release of pollutants is assumed to be proportional to the rainfall intensity and the pollutant mass available in the catchment. The treatment unit submodel is based on a serial tanks approach. The number of tanks is defined according of the hydraulic residence time and the geometric characteristics of the treatment unit, reproducing the hydraulic behaviour of the unit (including hydraulic short-circuiting). The fate of TSS and Cu is modelled by including the following processes among a larger range of processes represented in the STUMP model: settling and resuspension of sediments (TSS) and adsorption/desorption of Cu to/from TSS, thus representing Cu as both a particle-bound and a dissolved species. Settling and resuspension is modelled in a lumped manner for the whole treatment unit focusing solely on TSS and the sorbed Cu. Particle size distribution, effects of wind and temperature on resuspension, etc. are not considered by the model. The catchment submodel provides total pollutant loads and concentrations, while the treatment unit distinguishes between the dissolved and the particle-bound fractions. For more details regarding the model refer to Vezzaro et al. (2012a).

Model inputs include the rainfall time series and the pollutant fluxes released by sources in the catchment—identified by using land usage data stored in GIS databases [or inversely via uncertainty calibration, see Vezzaro et al. (2012b, 2015) for details]. Model parameters were estimated by using the measured rainfall data and the flow and quality data collected at the pond inlet and outlets during part of the monitoring period (May–October 2010, 6 runoff events).

### Rainfall time series used as input to scenario simulations

**Fig. 3:**
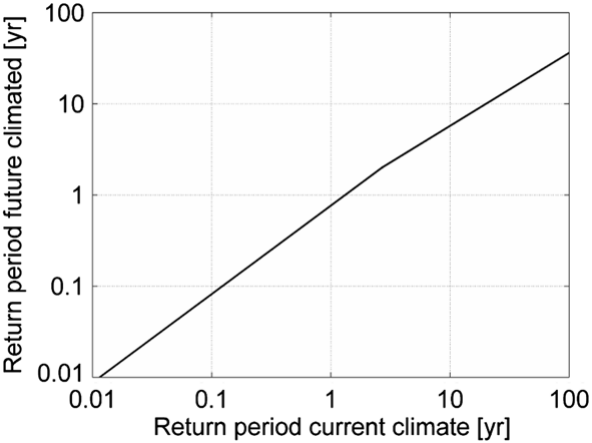
Relation between return periods of event volumes under current and future climate conditions

The study employed two 100-year synthetic rain series that represent climatic conditions in Denmark corresponding to the current climate and the future climate in year 2100, with the latter characterized by an increase in heavy precipitation volumes for all return periods higher than 0.1 years compared to the current situation (Fig. 3). The two rainfall series were constructed using an improved version of the Random Parameter Bartlett–Lewis Rectangular Pulse Model (Onof et al. 2000), which was initially calibrated using a generic Danish high-resolution rain series and emphasizing rainfall event peak intensities and volumes. Estimation of the potential increase of extreme precipitation was based on time series of precipitation derived from model simulations using the high-resolution Regional Climate Model (RCM) HIRHAM4 nested within the Global Circulation Model HadAM3H using the IPCC SRES scenario A2 as climate forcing (Christensen and Christensen 2009). A2 is a high-middle emission scenario used previously to assess potential climate change impacts on future extreme rainfall in Denmark (Larsen et al. 2009), and the RCM results were further downscaled to 100-year point estimates with a temporal resolution of 5 min by Onof and Arnbjerg-Nielsen (2009). Sunyer et al. (2014a) assessed climate change impacts on precipitation extremes using the much larger and more recent ENSEMBLES database (van den Linden and Mitchell 2009) based on the A1B scenario, and other yet unpublished studies by the same group are based on RCM output using the RCP4.5 forcing. The RCM data behind the downscaled times series by Onof and Arnbjerg-Nielsen (2009) are in accordance with other more recent simulations based on different assumptions about scenarios, which justifies that they are adequate for the purpose of this study, even though the simulated precipitation series have some biases compared with observed historical series, with respect to average properties of rainfall as reflected in e.g. mean annual precipitation and ADP.

As shown in Arnbjerg-Nielsen (2012) the used weather generator and downscaling method underestimates the impacts of climate change on rainfall extremes compared to analyzing output from RCMs directly, and thus a volumetric correction factor was applied to allow the extremes to have the right properties while still occurring at the right time of the year. The volumetric correction is event-based and uses the volume per event of current rainfall extremes described by Madsen et al. (2009). The correction ensures that the properties of extremes are close to those of the anticipated future, but at the cost of a correct annual average precipitation. The rain series should thus be used with caution if a high proportion of the loading is related to small events; the statistics of ADP can furthermore not be considered reliable and the statistical analysis in the following is therefore focused on extreme rainfall properties. Figure 3 shows the differences between the return periods of precipitation event volumes for the two synthetic rain series representing the current and anticipated future climates. A 100 year event in the current climate corresponds to a 40 year even in the future climate, a 10 year event in the current climate corresponds to a 6 year event in the future climate, and a 1 year event in the current climate corresponds to a 0.8 year event in the future climate, etc. This means that the impacts of climate change increases with increasing return period, as confirmed in general by literature sources (i.e. Arnbjerg-Nielsen et al. 2013).

It was assumed that the pollutant fluxes released by the sources in the catchment do not change due to climate changes, i.e. only changes in the rainfall patterns were responsible for the differences between the results of the two scenarios. Also, the size distribution of particles entering the stormwater system and the effects of temperature and wind on settling and resuspension (not included in the used model) were assumed to be unchanged (i.e. TSS settling properties were constant in the two scenarios).

## Results and discussion

### Current stormwater quality

Table 1 summarizes the data for 10 stormwater runoff and pond outlet events collected during the monitoring period. Outlet flow and concentration data was only available for 7 events. This may seem limited when aiming to quantify e.g. annual pollution runoff or pollution reduction efficiency. The model was calibrated using only the six first events. This may seem limited when using the results for assessing ecological impacts. We however aim here to explore the possible influence of potential climate change on stormwater runoff quality and treatment efficiency relative to the current situation, using a model calibrated to the current situation, and for this purpose this amount of data is sufficient.

**Table 1:** Main characteristics of the 10 events monitored in the period May 2010 to May 2011

Stormwater runoff event	1	2	3	4	5	6	7	8	9	10
Rain										
ADP (days)	4.0	0.3	1.7	1.4	11.5	1.6	0.4	2.2	6.8	5.2
Maximum 5-min intensity (mm/h)	1.33	48.0	7.68	7.31	6.40	4.80	2.88	5.6	2.88	54.0
Depth (mm)	2.2	6.4	7.6	11.6	17.6	3.0	7.4	2.2	14.0	7.4
Duration (h)	3.02	0.27	12.5	30.6	12.8	5.48	20.7	0.93	21.4	3.98
Inlet										
No. of samples	2	7	8	9	11	4	4	1	5	2
Flow max (L/s)	73	525	111	235	284	132	208	148	228	368
TSS max (mg/L)	100	1425	42	79	32	209	163	65	136	655
Cu_tot_ max (μg/L)	46	840	20	36	35	114	85	49	70	165
Cu_diss_ max (μg/L)	25	16	20	13	14	22	66	28	30	10
Outlet										
No. of samples	0	0	0	13	2	1	7	1	4	1
Flow max (L/s)	–	–	–	114	126	63	94	58	96	98
TSS max (mg/L)	–	–	–	22	11	14	30	17	26	39
Cu_tot_ max (μg/L)	–	–	–	14	16	20	28	12	19	17
Cu_diss_ max (μg/L)	–	–	–	7	8	15	19	10	12	6

Inlet and outlet data from the pond are maximum event values for stormwater runoff events as defined on Fig. 2 [flow (2 min resolution), TSS, and total and dissolved Cu]. Rainfall data (ADP, maximum 5 min intensity, depth, duration) are given using the same event definition, i.e. in some cases several individual rainfall events are lumped into one

–, not measured

**Fig. 4:**
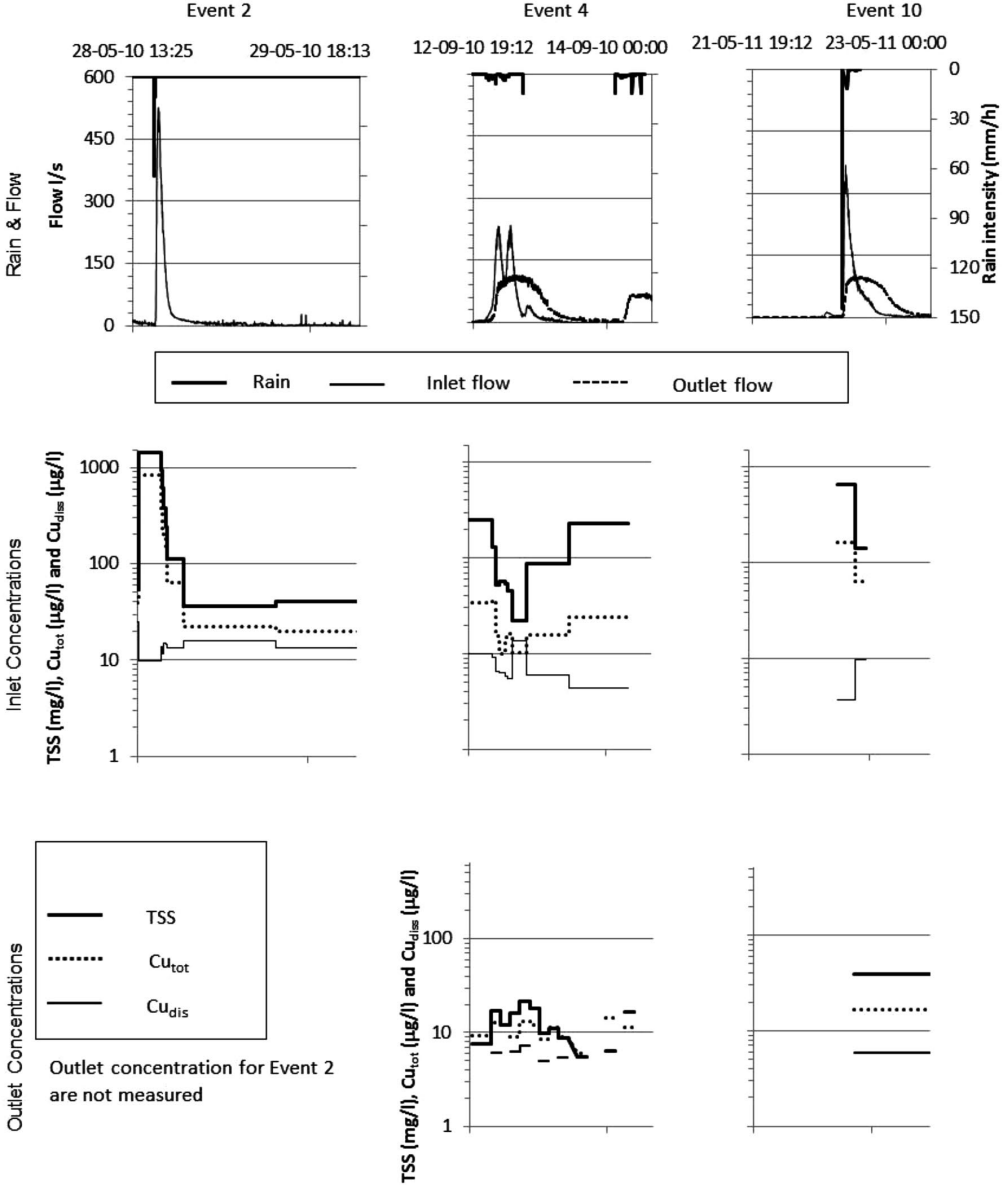
Dynamics of flow, TSS, and total and dissolved Cu concentrations in the pond inlet and outlet during stormwater runoff events 2, 4 and 10. The step-curves for inlet and outlet concentrations illustrate the sampling intervals

The 10 runoff events correspond to return periods ranging from very frequent and up to 1 year return period based on their intensity and/or volume. Concentrations of TSS and Cu_tot_ were highest for high-intensity precipitation events, most likely due to erosion of sediments accumulated on the catchment surfaces or in the pipe and channel system in the catchment. Figure 4 shows the temporal dynamics of 3 selected stormwater runoff and pond outlet events (Events 2, 4 and 10). The observed inlet concentrations of TSS, Cu_tot_ and Cu_diss_ were in the ranges 6–1425 mg/L, <5–840 μg/L, and <5–66 μg/L respectively. The Cu_diss_/Cu_tot_ ratio varied from event to event and during events and was in the range of <0.1–1. The ratio was lowest in the beginning of the stormwater runoff events and increased during the events, meaning that particulate bound copper dominated the total concentration in the beginning of events and dissolved copper dominated hereafter. Tuccillo (2006) also reported that the partitioning of Cu varied between storms and sites and reported a similar range of Cu_diss_/Cu_tot_. Each rain event resulted in a distinct flow peak whereas concentration peaks for TSS and Cu were only observed for the first rain event during a storm water runoff event. These results are in accordance with other studies (e.g. Deletic and Maksimovic 1998).

The observed concentrations of TSS, Cu_tot_ and Cu_diss_ in the pond discharge were in the ranges 2–39 mg/L, <5–28 μg/L, and <5–19 μg/L respectively. The TSS and Cu_tot_ concentration levels were similar to those measured in other studies of stormwater pond systems (e.g. Wium-Andersen et al. 2011; Vezzaro et al. 2012a, b; Carpenter et al. 2014). The range for Cu_diss_/Cu_tot_ was <0.3–1 and varied from event to event and during events, like for the inlet.

The concentrations in the stormwater runoff (i.e. the concentrations at the pond inlet) and in the outlet from the pond showed that a substantial amount of the particulate matter was retained in the stormwater retention pond and that the removal of dissolved Cu was lower than the total Cu removal. This is in accordance with previous studies, indicating that settling of the particles is the main removal mechanism in stormwater retention ponds (e.g. Scholes et al. 2008).

**Fig. 5:**
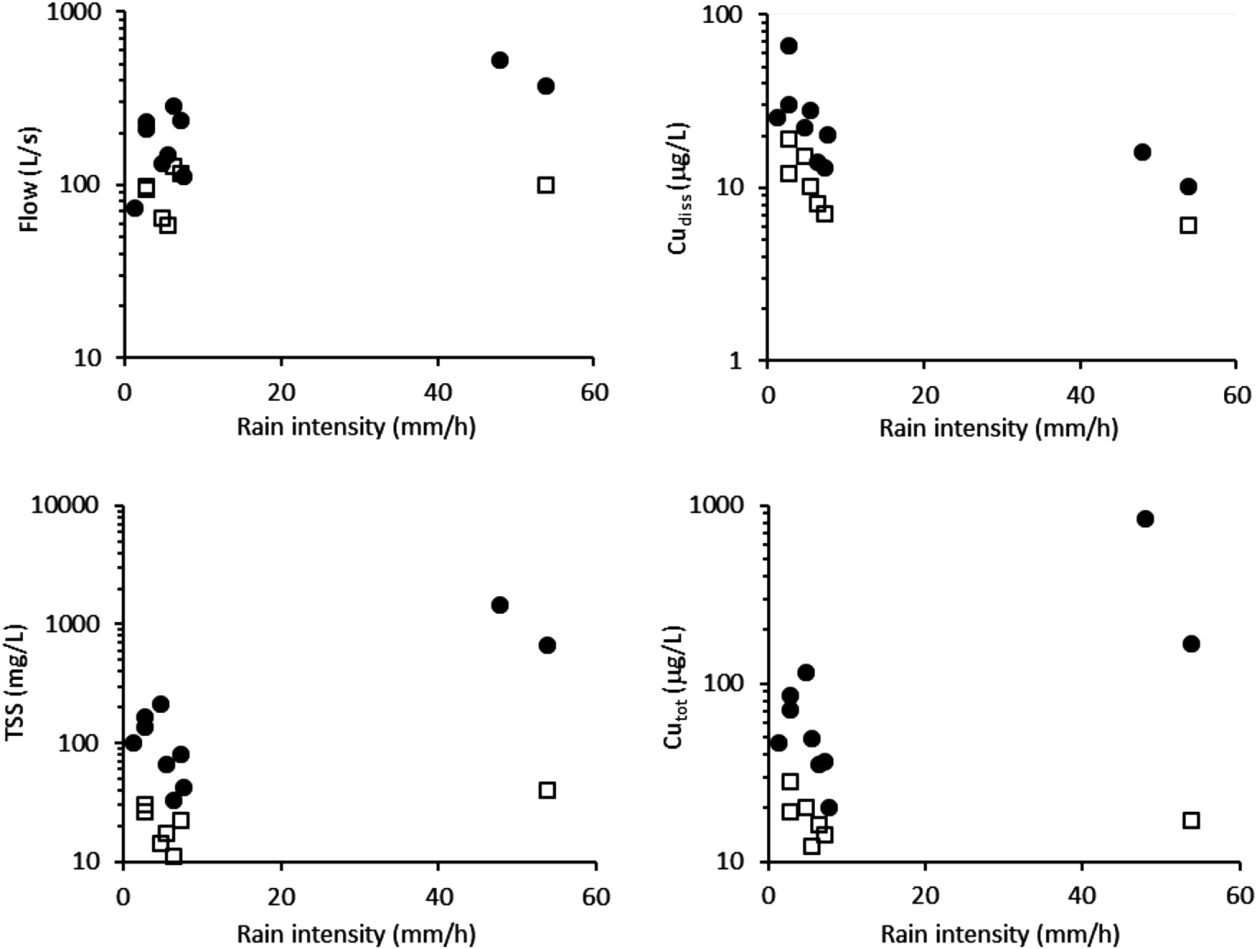
Effect of peak event rain intensity on maximum event values for flow (*top left*), Cu_diss_ (*top right*), TSS (*bottom left*) and Cu_tot_ (*bottom right*) in the stormwater runoff (*bullets*) and pond outlet (*hollow squares*) events

Figure 5 shows the effect of peak event rain intensity on the maximum event values for flow, TSS, Cu_tot_ and Cu_diss_ in the inlet and outlet from the stormwater retention pond. The highest rain intensities (48 and 54 mm/h) were measured during stormwater runoff event 2 and 10, which also resulted in the highest inlet flows and TSS and Cu_tot_ concentrations. Regression analyses showed that higher rain intensities generally resulted in higher flow, TSS and Cu_tot_ in the inlet to the stormwater retention pond (p < 0.05, R^2^ in the range 0.5–0.7), underlining the importance of high intensity rain events in the estimation of the total load to the pond. These findings are similar to those reported in the literature (e.g. Borris et al. 2013; Deletic and Maksimovic 1998; He et al. 2010). The rain intensity did however not show any effect on flow, TSS and Cu_tot_ in the outlet from the pond (p > 0.05, R^2^ in the range 0.0–0.5). The maximum Cu_diss_ concentrations in both inlet and outlet samples were also unaffected by the rain intensities (p > 0.05, negative slopes with R^2^ in the range 0.2–0.3).

Table 1 shows main characteristics of the 10 events monitored during the period May 2010 to May 2011. Maximum inlet and outlet data from the pond for stormwater runoff events as defined on Fig. 2 [flow (2 min resolution), TSS, and total and dissolved Cu]. Rainfall data (ADP, maximum 5 min intensity, depth, duration) are given using the same event definition, i.e. in some cases several individual rainfall events are lumped into one.

### Expected changes in pollution loads and extreme concentrations due to climate change

**Fig. 6:**
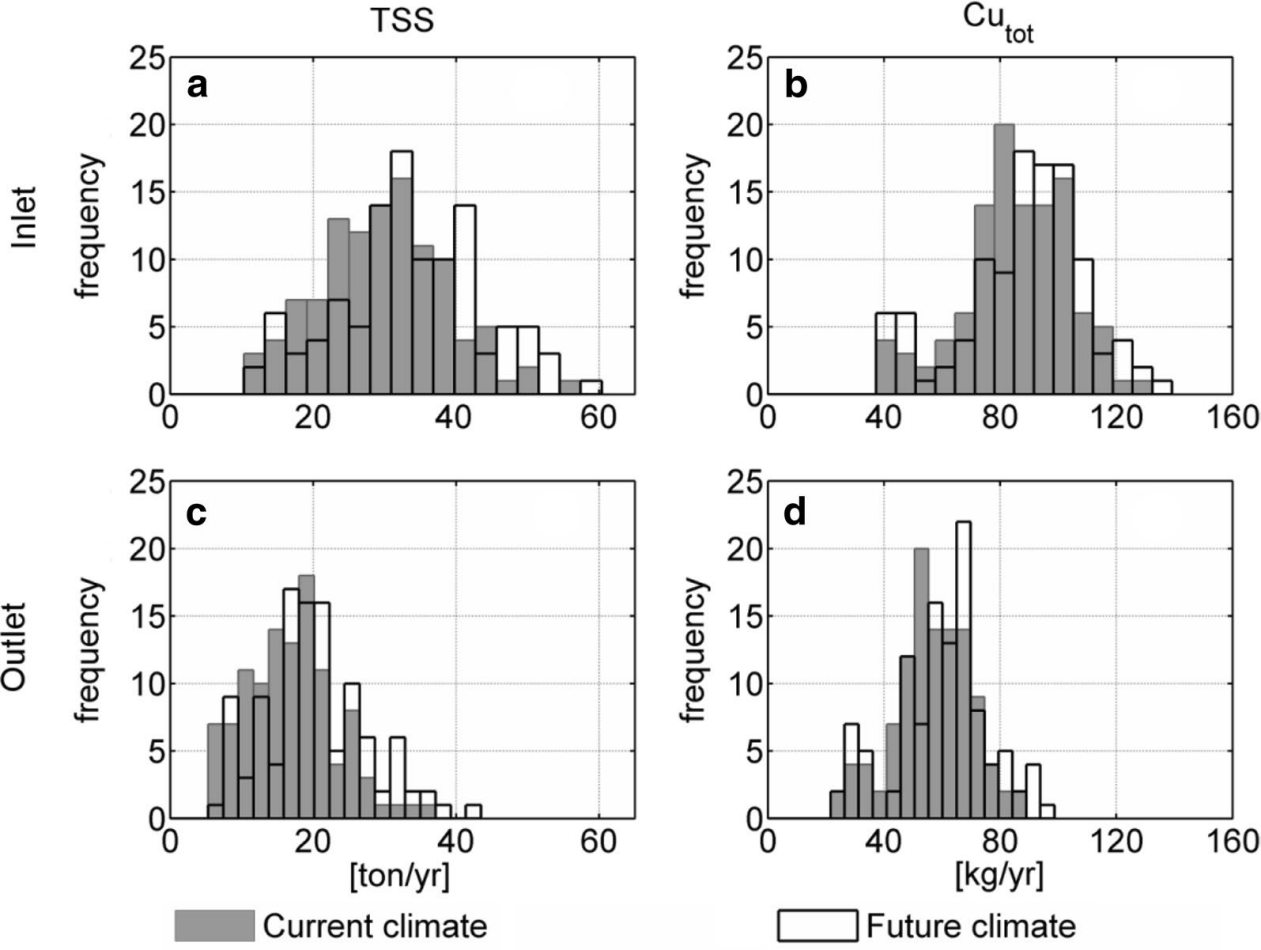
Histograms of simulated current and future climate yearly loads of TSS (**a**, **c**) and Cu_tot_ (**b**, **d**) at the pond inlet (**a**, **b**) and outlet (**c**, **d**)

**Fig. 7:**
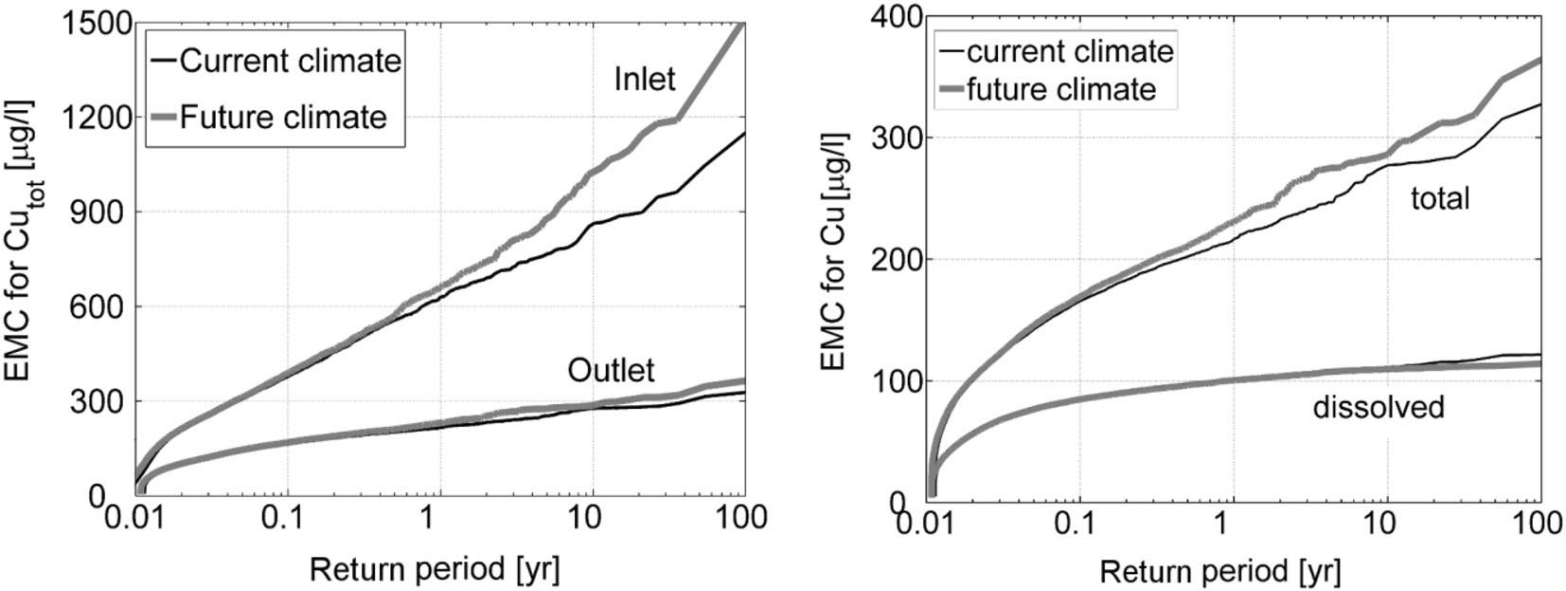
Simulated current and future climate Cu EMCs ranked according to return period. Comparison of (*left*) total EMCs in the pond inlet and (*right*) total and dissolved EMCs at the pond outlet

The expected effect of climate change was evaluated based on 2 statistics computed from the output from the simulation model: yearly loads (Fig. 6) and extreme statistics of EMCs (Fig. 7).

The simulated yearly loads of TSS and Cu_tot_ discharged from the catchment were increased by climate change (the median yearly load increased 12% for TSS and 6% for Cu—Fig. 6, top). An important factor to consider when looking at these results is the assumption of constant pollutant fluxes released to the catchment in the two scenarios, i.e. the total mass entering the catchment did not change from the current to the future climate scenario. When looking at the event loads (not shown) an increase in the number of small frequent events (less than 100 g/event) and yearly loads were noticed for Cu.

An increase in the yearly total loads of TSS and Cu_tot_ discharged from the pond outlet was noticed for the simulated pollutants (the median yearly load increased 14% for TSS and 7% for Cu—Fig. 6, bottom). This increase is directly connected to the higher particle loads at the inlet to the pond, and to a modification of the flow entering the pond resulting in a lower HRT, resuspension of sediments and increase of the hydraulic short-circuiting of the pond. Nevertheless, the removal performance of the pond remained in the same order of magnitude (with a median value for the yearly removal rate of 33.9% for the current scenario and 32.5% for the climate change scenario for Cu_tot_).

Figure 7 shows the simulated Cu EMCs against their estimated return periods. The EMC values for Cu_tot_ at the pond inlet (Fig. 7 left) increased for events with return periods larger than 0.5 years. For example, a 10 year event in the current climate corresponds to a 4 year event in the future climate and an event exceeded once per year in the current climate will be exceeded twice per year in the future. Direct comparison of the dissolved concentration output to ambient water quality standards will require a more elaborate calibration of the model considering more monitoring data as well as prediction uncertainty. Furthermore, the total concentrations in the water phase simulated here cannot be directly compared to toxic sediment phase concentrations given in literature or regulations, but similar effects of climate change have been observed by other authors. For example, Wilson and Weng (2011) showed for the Des Plains River watershed, Illinois, that climate change will result in higher total suspended solids (TSS) loads during late winter and early spring, while the reverse trend will be the case for summer periods. He et al. (2011) simulated the effect of climate change on 16 rain events. They showed that the EMCs for turbidity (indicator of particulate solids) increased for all storm events except for 3 events of short duration (<1 h), where the turbidity decreased due to climate change. They explained the observed effect on turbidity as a combination of duration and intensity influencing the ratio of re-suspension of solids from sewers and wash-off of suspended solids from the land surface.

Increased Cu_tot_ outlet concentrations were also observed for events with return periods greater than 0.5 years (Fig. 7 left, and with different vertical scale on Fig. 7 right). The increase in the pond inlet and outlet concentrations can be related to the change in the rainfall patterns, i.e. the increase in extreme rainfall intensities for large rain storms is reflected in the greater pollutant fluxes per event for large return periods. The results however showed only a very minor effect of climate change on the dissolved fraction (Fig. 7, right). Therefore, the observed increase in outlet Cu_tot_ for the future climate scenario compared to the current climate scenario was mainly due to an increase in the particulate fraction discharged from the pond. As the higher climate-change related flows affected only processes strongly related to particles (settling/resuspension) and the particle settling characteristics did not change due to longer ADPs in the future scenario, the concentrations of the dissolved fraction were not affected by climate change (as suggested by the two almost overlapping lines in Fig. 7, right).

### Implications of the results

By looking at the different behaviour of the total inlet and outlet concentrations in the two climate scenarios (Fig. 7 left), the model suggests that the existing retention pond attenuates the climate change signal, i.e. the change of the extreme pond outlet EMCs was smaller than the change of the extreme pond inlet EMCs. Furthermore, since the acute toxic effects are mainly caused by dissolved metals, climate change is not expected to have a substantial effect on the acute toxicity impacts caused by dissolved metals.

The results presented here were based on yearly load averages and extreme statistics for EMCs, and seasonality was not taken into account. The performance of the pond may however be different in summer and wintertime: the runoff model, in fact, does not consider snow and snowmelt in the catchment, and the pond model does not consider the presence of ice or influence of temperature on the removal of TSS and heavy metals. Potential changes in particle size distributions caused by resuspension of bigger particles due to higher peak rainfall intensity in the future were furthermore not considered; particle size distributions were not measured in the current study and are not addressed by the employed model. Further experimental research and more complex models are needed to address these specific issues and to provide a better analysis of the potential impacts of climate change on the performance of stormwater retention ponds. The statistics of ADP in the synthetic rainfall time series used here could not be considered sufficiently reliable to allow a detailed interpretation, and further work on generating time series for use in studies like this is therefore needed.

Stormwater runoff contains many more pollutants than those studied herein. Eriksson et al. (2007) list 25 representative stormwater priority pollutants that may be considered when evaluating chemical risks related to stormwater management strategies, including general water quality parameters (organic matter, TSS, nutrients and pH), and a range of heavy metals, polyaromatic hydrocarbons (PAH), herbicides and other representative industrially derived compounds. Several other heavy metals (Cd, Ni, Zn) and phosphorus are also found partly associated with particles in stormwater runoff and are therefore expected to be affected by settling inhibition for large rain storms, as documented for Cu in this study. The same applies to some of the organic compounds (e.g. glyphosate and pyrene, Vezzaro et al. 2011).

Retention ponds like the one studied here are abundant in Denmark and across the world. Although they typically treat stormwater runoff from areas of limited size, larger urban catchments typically contain many sub-catchments with retention ponds that react like the one studied in this paper. There may thus be cumulative negative effects from multiple catchments, which should be taken into account when planning future emission control strategies to protect surface water resources surrounding urban areas. The results presented here can be transferred to other systems with similar catchment characteristics (including land use and catchment imperviousness), pond volume to catchment area ratio and hydraulic retention time (as defined in Persson et al. 1999).

## Conclusions

This study illustrated how the anticipated climate change impacts on extreme rainfall in Denmark may potentially lead to changes in stormwater runoff quality and removal efficiency of a stormwater retention pond. Measurements of hydrology and water quality of the current system showed the importance of extreme events for high suspended solids and particulate Cu concentrations and for the estimation of the total load discharged from the catchment. Model simulation results suggested that the future climate scenario, characterized by more intense events, can result in increased total event mean concentrations in runoff from the catchment for return periods above 0.5 years. A smaller effect was found for discharge from the pond, suggesting that the pond attenuated the climate change signal. A minor (7–14%) increase of total yearly loads discharged from the retention pond was furthermore found, due to the increasing inlet loads coming from the catchment and a minor inhibition of the particle removal processes due to higher peak inlet flows. This tendency was, however, not seen for dissolved Cu, which is not affected by settling processes and is the major cause of aqueous toxicity. Thus the potential increase in toxicity of the pond discharges due to climate change is expected to be minimal. Similar results are expected for other particle bound metals and slowly biodegradable organic substances such as PAH. This may be important to consider for the many stormwater retention ponds existing in Denmark and across the world where similar climate change effects are expected. Further work is needed to investigate the effect of potential increased antecedent dry periods and thus higher extreme pollution loads due to climate change, using synthetic time series constructed with this purpose in mind. Furthermore, more elaborate monitoring campaigns and model calibrations considering the involved prediction uncertainty are needed to investigate if the effects of climate change will challenge the ambient water quality standards in use today.
